# Hospital Outcomes of Community-Acquired SARS-CoV-2 Omicron Variant Infection Compared With Influenza Infection in Switzerland

**DOI:** 10.1001/jamanetworkopen.2022.55599

**Published:** 2023-02-15

**Authors:** Lea Portmann, Marlieke E. A. de Kraker, Georg Fröhlich, Amaury Thiabaud, Maroussia Roelens, Peter W. Schreiber, Nicolas Troillet, Anne Iten, Andreas Widmer, Stephan Harbarth, Rami Sommerstein

**Affiliations:** 1 Department of Health Sciences and Medicine, Clinic St Anna, University of Lucerne, Lucerne, Switzerland; 2 Geneva University Hospitals and Faculty of Medicine, Infection Control Program and WHO Collaborating Center, Geneva, Switzerland; 3 Heart Clinic Lucerne, Lucerne, Switzerland; 4 Charité-Universitätsmedizin Berlin, Berlin, Germany; 5 Institute of Global Health of the University of Geneva, Geneva, Switzerland; 6 Division of Infectious Diseases and Hospital Epidemiology, University Hospital Zürich and University of Zürich, Zürich, Switzerland; 7 Department for Infectious Diseases, Central Institution, Valais Hospital, Sion, Switzerland; 8 Swissnoso, the National Center for Infection Control, Bern, Switzerland; 9 Department for Infectious Diseases, University Hospital Basel, Basel, Switzerland; 10 Department of Infectious Diseases, Bern University Hospital, Bern, Switzerland

## Abstract

**Importance:**

With the ongoing COVID-19 pandemic, it is crucial to assess the current burden of disease of community-acquired SARS-CoV-2 Omicron variant in hospitalized patients to tailor appropriate public health policies. Comparisons with better-known seasonal influenza infections may facilitate such decisions.

**Objective:**

To compare the in-hospital outcomes of patients hospitalized with the SARS-CoV-2 Omicron variant with patients with influenza.

**Design, Setting, and Participants:**

This cohort study was based on a national COVID-19 and influenza registry. Hospitalized patients aged 18 years and older with community-acquired SARS-CoV-2 Omicron variant infection who were admitted between January 15 and March 15, 2022 (when B.1.1.529 Omicron predominance was >95%), and hospitalized patients with influenza A or B infection from January 1, 2018, to March 15, 2022, where included. Patients without a study outcome by August 30, 2022, were censored. The study was conducted at 15 hospitals in Switzerland.

**Exposures:**

Community-acquired SARS-CoV-2 Omicron variant vs community-acquired seasonal influenza A or B.

**Main Outcomes and Measures:**

Primary and secondary outcomes were defined as in-hospital mortality and admission to the intensive care unit (ICU) for patients with the SARS-CoV-2 Omicron variant or influenza. Cox regression (cause-specific and Fine-Gray subdistribution hazard models) was used to account for time-dependency and competing events, with inverse probability weighting to adjust for confounders with right-censoring at day 30.

**Results:**

Of 5212 patients included from 15 hospitals, 3066 (58.8%) had SARS-CoV-2 Omicron variant infection in 14 centers and 2146 patients (41.2%) had influenza A or B in 14 centers. Of patients with the SARS-CoV-2 Omicron variant, 1485 (48.4%) were female, while 1113 patients with influenza (51.9%) were female (*P* = .02). Patients with the SARS-CoV-2 Omicron variant were younger (median [IQR] age, 71 [53-82] years) than those with influenza (median [IQR] age, 74 [59-83] years; *P* < .001). Overall, 214 patients with the SARS-CoV-2 Omicron variant (7.0%) died during hospitalization vs 95 patients with influenza (4.4%; *P* < .001). The final adjusted subdistribution hazard ratio (sdHR) for in-hospital death for SARS-CoV-2 Omicron variant vs influenza was 1.54 (95% CI, 1.18-2.01; *P* = .002). Overall, 250 patients with the SARS-CoV-2 Omicron variant (8.6%) vs 169 patients with influenza (8.3%) were admitted to the ICU (*P* = .79). After adjustment, the SARS-CoV-2 Omicron variant was not significantly associated with increased ICU admission vs influenza (sdHR, 1.08; 95% CI, 0.88-1.32; *P* = .50).

**Conclusions and Relevance:**

The data from this prospective, multicenter cohort study suggest a significantly increased risk of in-hospital mortality for patients with the SARS-CoV-2 Omicron variant vs those with influenza, while ICU admission rates were similar.

## Key Points

**Question:**

Are hospital outcomes of SARS-CoV-2 variant B1.1.529 (SARS-CoV-2 Omicron variant) comparable with outcomes of seasonal influenza infections among hospitalized patients?

**Findings:**

In this cohort study of 5212 patients hospitalized with the SARS-CoV-2 Omicron variant or influenza A or B in Switzerland, the SARS-CoV-2 Omicron variant was associated with an approximately 1.5-fold higher risk of in-hospital all-cause mortality up to day 30 compared with influenza.

**Meaning:**

These findings suggest that, despite virus evolution and improved management strategies, patients with the SARS-CoV-2 Omicron variant had a higher risk of in-hospital mortality than those with influenza.

## Teaser

This cohort study compares in-hospital outcomes among patients hospitalized with the SARS-CoV-2 Omicron variant vs those hospitalized with influenza A or B in Switzerland.

## Introduction

Since the beginning of the COVID-19 pandemic in 2019, several variants of SARS-CoV-2 have emerged that differ in virulence and transmission rate.^1^ The dominant variant during the period from January 15 to March 15, 2022, in Switzerland was B.1.1.529 (Omicron), which was responsible for more than 95% of all sequenced COVID-19 infections in Switzerland.^2^ Omicron was first reported in South Africa in November 2021 and was designated as a variant of concern by the World Health Organization on November 26.^3^ Multiple studies have shown that the Omicron variant is associated with a lower risk of hospitalization and death than the Delta variant.^1,4,5,6,7,8,9^ However, the Omicron variant has a higher transmission rate than previous variants.^10^ Vaccine effectiveness against symptomatic disease caused by the Omicron variant appears to be lower than that caused by the Delta variant.^11^ Nevertheless, especially after a booster, vaccination reduces the risk of required hospitalization and death.^1^ The vaccines available in Switzerland were mostly mRNA-1273 (Moderna) or BNT162b2 (Pfizer/BioNTech). By January 2022, more than 9 million doses of mRNA-1273 and more than 5 million doses of BNT162b2 had been administered. This represents an immunization coverage of approximately 69%, and approximately 55% had a vaccination in the last 6 months.^2^

Seasonal influenza is being monitored by the Sentinella Reporting System of the Federal Office of Public Health. Within this scope, every year the circulating subtypes are determined. From 2018 to 2022, mostly influenza A was circulating, while influenza B was a minority.^12^ The subtypes A (H3N2) and A (H1N1) circulated at similar levels during the 2018 to 2022 period combined. In 2022, subtype A (H3N2) was dominant, accounting for 94% of all sequenced samples. Influenza vaccination is only recommended for high-risk groups in Switzerland. In 2018 to 2022, the vaccination rates varied between 28% and 38% in patients older than 65 years.^12^ There are similarities between COVID-19 and influenza, 2 viruses that mainly affect the lungs, resulting in upper respiratory tract infection symptoms, such as cough, runny nose, sore throat, fever, headache, and fatigue. Both can be fatal and are easily transmitted by respiratory particles.^13^ However, several studies have examined hospital outcomes of COVID-19 compared with influenza and found that COVID-19 is associated with a higher risk of death, extrapulmonary organ dysfunction, and higher health care resource use.^14,15,16^

In our previous study, we reported a 2-fold to 3-fold higher rate of death and ICU admissions between community-acquired COVID (wild type and Alpha) vs influenza.^16^ A comparison of outcomes among patients hospitalized with the Omicron variant and those hospitalized with influenza was performed only in unvaccinated children so far, and it was found that Omicron was associated with a higher risk of ICU admission than influenza.^17^

Because the Omicron variant appears to be associated with a less severe outcome, it is important to understand whether in-hospital outcomes of this COVID-19 variant have become comparable with other frequent viral airway infections, like seasonal influenza A or B infections.^9^ We therefore analyzed a large, nationwide database to compare mortality and ICU admission rates among hospitalized Swiss patients with the SARS-CoV-2 Omicron variant vs influenza.

## Methods

### Ethical Statement

This cohort study was approved by the Ethics Committee of the Canton of Geneva, Switzerland. Data collection was approved by all local ethics committees. Because all data were deidentified, informed consent was not required. Studies were designed, conducted, and reported in accordance with the 2013 version of the Declaration of Helsinki.^18^ This report follows the Strengthening the Reporting of Observational Studies in Epidemiology (STROBE) reporting guidelines.^19^

### Study Design, Setting, and Participants

This is a retrospective multicenter cohort study of patients hospitalized with the SARS-CoV-2 Omicron variant or influenza A or B in Switzerland based on a prospective, national registry. Overall, 13 hospitals (including all 5 university, large cantonal, and private hospitals) collected data on the SARS-CoV-2 Omicron variant and influenza, while 2 smaller hospitals collected data for either the SARS-CoV-2 Omicron variant or influenza only. To be eligible for the study, patients had to be aged 18 years or older and have the SARS-CoV-2 Omicron variant or influenza infection proven by a positive polymerase chain reaction test or a positive antigen test. Patients with the SARS-CoV-2 Omicron variant were included if they were admitted in a participating center between January 15 and March 15, 2022. During this period, more than 95% of sequenced COVID-19 infections were due to the Omicron B.1.1.529 variant.^2^ Influenza data were included from January 1, 2018, to March 15, 2022. Patients without a completed follow-up form on August 30, 2022, were censored.

### Data Sources

All data were collected as part of the hospital-based surveillance of the SARS-CoV-2 Omicron variant and influenza in Switzerland. This database was originally initiated by the Institute of Global Health and the Infection Control Program at the University of Geneva. Data were collected using an electronic form created in REDCap. Outcome data were collected from the same sources in the same manner. Data included patient demographic characteristics; vaccination information; main comorbidities; ICU admission and discharge dates; utilization of noninvasive or invasive ventilation; complications, such as pulmonary, cardiovascular, neurological and kidney disease; and dates of hospital admission and discharge or death.

### Outcome Measures

The primary outcome was all-cause in-hospital mortality. The secondary outcome was admission to the ICU. Further exploratory end points include ventilation, pulmonary complications (acute respiratory distress syndrome and/or pneumonia), cardiovascular complications, kidney complications, neurological complications, length of hospital stay, and antibiotic treatment.

### Statistical Analysis

Statistical analysis was performed as described before.^16^ In short, categorical data were tested with the Pearson χ^2^ test and ordinal data with the Mann-Whitney U test.^16^ The cause-specific Cox hazard model was used for primary and secondary outcomes to account for competing risks and prevent overestimation of the outcomes of COVID-19.^16,20^ Competing risks were hospital discharge for hospital mortality and discharge and death before ICU admission for ICU admission. Patients who stayed longer than 30 days were right-censored.^16^ To account for differences in baseline characteristics, inverse probability weighting was performed.^16^ For this, a propensity score analysis was performed using clinical and epidemiological factors that were present at admission and significantly associated with influenza or the SARS-CoV-2 Omicron variant as determined by univariate logistic regression models. To evaluate appropriateness of this analytic approach, a validation of the weights and proportional hazard assumption was performed (eTables 1 and 2 in Supplement 1). For the main analyses, weights were applied for sex, age, and university hospital as treatment center. The inclusion date for the survival analysis was the date of hospital admission. An additional survival analysis was performed using the date of positive test result as the inclusion date. Truncation at 1st and 99th percentile was applied. In a second step, subdistribution hazard analysis was performed using the Fine-Gray model to determine the cumulative rate of death or ICU admission associated with the SARS-CoV-2 Omicron variant or influenza.^21^

All analyses were performed in R version 4.20 (R Project for Statistical Computing) using the packages survival, ipw, CausalGAM, and cmprsk. Two-tailed tests were performed, and *P* < .05 was considered statistically significant.^16^

A subgroup analysis was performed that focused on patients with COVID-19 or influenza who were hospitalized primarily because of (rather than with) their respective illness. To be classified as hospitalized because of COVID-19 or influenza, the reason for hospitalization had to include symptoms due to COVID-19 or influenza or decompensation of a chronic disease, evidently caused by COVID-19 or influenza, eg, a cardiac decompensation in the context of COVID-19 or influenza. Because this definition was only recently added to the surveillance system, analysis was restricted to patients from 2022. Additionally, a similar analysis (admitted because of COVID-19 or influenza) was performed with patients admitted during the same period. A second subgroup analysis was performed that only included patients with the SARS-CoV-2 Omicron variant who were unvaccinated (while information on influenza vaccination was largely missing). In another subgroup analysis, patients with an origin other than domicile were excluded.

## Results

### Baseline Characteristics

**Figure 1.**
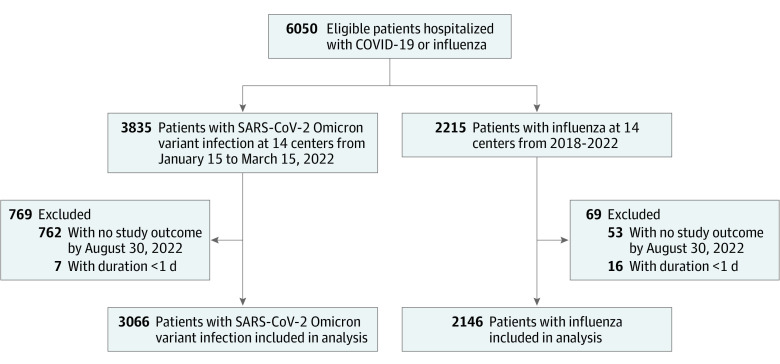
Study Flowchart

Of 6050 eligible patients, 5212 (86.1%) were included in the study; 769 (12.7%) were excluded with the SARS-CoV-2 Omicron variant and 69 (1.1%) with influenza (mainly because there was no follow up by August 30, 2022) (Figure 1). Overall, 3066 patients (58.8%) had the SARS-CoV-2 Omicron variant, and they were younger (median [IQR] age, 71 [53-82] years) than the 2164 patients (41.2%) with influenza (median [IQR] age, 74 [59-83] years; *P* < .001). Patients with the SARS-CoV-2 Omicron variant were less often female (1485 [48.4%]) than patients with influenza (1113 [51.9%]; *P* = .02). The 2146 patients with influenza consisted of 2064 (96.2%) with influenza A and 82 (3.8%) with influenza B. Of the patients with the SARS-CoV-2 Omicron variant, 1592 (51.9%) were vaccinated at least once and 773 (25.2%) had received 3 doses of vaccine. Of 469 sequenced COVID-19–positive samples, 446 (95.7%) were typed as Omicron. A detailed compilation of the different variants can be found in eTable 3 in Supplement 1. A detailed description of the baseline characteristics for patients with the SARS-CoV-2 Omicron variant and Influenza, including host, epidemiologic, and virologic features is shown in Table 1. Most patients with the SARS-CoV-2 Omicron variant (2743 [90.1%]) and influenza (1932 [90.0%]) were referred directly to the hospital from home. Any preexisting comorbidities were reported in 2295 patients with COVID-19 (79.5%) and in 1648 patients with influenza (80.9%; *P* = .22). Respiratory comorbidities were less frequently reported in patients with the SARS-CoV-2 Omicron variant (375 [13.1%]) than in patients with influenza(499 [24.6%]; *P* < .001). eTable 4 in Supplement 1 provides an overview of the CURB-65 severity score (confusion, uremia, respiratory rate, blood pressure, age ≥65 years) at admission.^22^

**Table 1.** Baseline Characteristics of 5212 Patients With the SARS-CoV-2 Omicron Variant Admitted from January 15 to March 15, 2022, or With Influenza A or B, Admitted from January 1, 2018, to March 15, 2022, in Switzerland

Characteristic	Patients, No. (%)		*P* value
	SARS-CoV-2 Omicron variant (n = 3066)	Influenza A or B (n = 2146)	
Age, median (IQR), y	71 (53-82)	74 (59-83)	<.001
Sex			
Female	1485 (48.4)	1113 (51.9)	.02
Male	1581 (51.6)	1033 (48.1)	
Admission to university hospital	1093 (35.6)	1347 (62.8)	<.001
BMI, median (IQR)	25.5 (22.4-29.3)^a^	25.3 (21.8-29.5)^b^	.16
COVID-19 vaccination status^c^			
Overall	1592 (51.9)		NA
With 2 doses	618 (20.2)	NA	
With 3 doses	773 (25.2)	NA	
Influenza vaccination status^d^	NA	215 (10.0)	NA
Sequenced samples COVID-19			
Overall	469 (15.3)	NA	NA
Omicron	449 (95.7)	NA	NA
Others	20 (4.3)	NA	NA
Virus type influenza			
A	NA	2064 (96.2)	NA
B	NA	82 (3.8)	NA
Treatment with oseltamivir	NA	1394 (65)	NA
Year of admission			
2018	NA	53 (2.5)	NA
2019	NA	985 (45.9)	NA
2020	NA	407 (19.0)	NA
2021	NA	42 (2.0)	NA
2022	3066 (100)	659 (30.7)	NA
Origin prior to hospitalization^e^			
Domicile	2743 (90.1)	1932 (90.0)	<.001
LTC facility	103 (3.4)	105 (4.9)	
Other hospital	153 (5.0)	53 (2.5)	
Other	45 (1.5)	56 (2.6)	
Comorbidities^f^			
Any	2295 (79.5)	1648 (80.9)	.22
Diabetes	546 (19.0)	451 (22.2)	.007
Chronic cardiovascular disease	1018 (35.5)	747 (36.7)	.40
Chronic kidney disease	606 (21.1)	368 (18.1)	.01
Chronic pulmonary disease	375 (13.1)	499 (24.6)	<.001
Chronic neurologic impairment	306 (10.7)	292 (14.4)	<.001
Hematological disorder	95 (3.3)	182 (8.9)	<.001
Chronic liver disease	100 (3.5)	92 (4.5)	.07

Abbreviations: BMI, body mass index (calculated as weight in kilograms divided by height in meters squared); LTC, long-term care; NA, not applicable.

^a^ Data missing for 581 patients (18.9%).

^b^ Data missing for 656 patients (30.6%).

^c^ Data missing for 418 patients (13.6%).

^d^ Data missing for 1664 patients (77.5%).

^e^ Data missing for 20 patients with COVID-19 (0.7%).

^f^ Data missing for between 5.2% and 7.2% of patients, mainly because 1 center did not report comorbidities.

### Outcomes

Table 2 provides an overview of the crude clinical outcomes. With the SARS-CoV-2 Omicron variant, 214 patients (7.0%) died during hospitalization, compared with 95 patients (4.4%) in the influenza group (*P* < .001). Hospital stay for patient with the SARS-CoV-2 Omicron variant was statistically shorter (median [IQR], 6 [3-10] days) than for patients with influenza (median [IQR], 6 [4-12] days; *P* < .001).

**Table 2.** Unadjusted Crude Outcomes of 5212 Patients With the SARS-CoV-2 Omicron Variant or Influenza A or B in Switzerland

Outcome	Patients, No. (%)		*P* value
	SARS-CoV-2 Omicron variant (n = 3066)	Influenza A/B (n = 2146)	
In-hospital deaths	214 (7.0)	95 (4.4)	<.001
Admission to the ICU	250 (8.6)^a^	169 (8.3)^b^	.79
Length of ICU stay, median (IQR), d	3 (1-11)	4 (2-8.5)	.71
Invasive ventilation in ICU	115 (46.4)	77 (46.1)	>.99
Length of hospital stay, median (IQR), d	6 (3-10)	6 (4-12)	<.001
Complications^c^	1679 (57.4)	1365 (67.3)	<.001
Respiratory complications	1237 (42.5)	1142 (56.3)	<.001
Cardiac disease	238 (8.5)	310 (15.3)	<.001
Neurologic impairment	70 (2.5)	110 (5.4)	<.001
Kidney impairment	267 (9.5)	232 (11.4)	.03
Antibiotic treatment	626 (21.8)	1160 (57.1)	<.001

Abbreviation: ICU, intensive care unit.

^a^ Data missing for 146 patients (4.8%).

^b^ Data missing for 113 patients (5.3%).

^c^ Data missing for between 4.4% and 8.5% of patients, mainly because 1 center did not report complications.

The need for ICU admission was similar in both groups: 250 patients with the SARS-CoV-2 Omicron variant (8.6%) and 169 with influenza (8.3%; *P* = .79). Duration of stay in the ICU was not different for patients with the SARS-CoV-2 Omicron variant (median [IQR], 3 [1-11] days) and for patients with influenza (median [IQR] 4 [2-9] days; *P* = .71). Overall, complications were reported less frequently in patients with the SARS-CoV-2 Omicron variant (1679 [57.4%]) than in patients with influenza (1365 [67.3%]; *P* < .001). A detailed comparison of additional complications is provided in Table 2.

**Figure 2.**
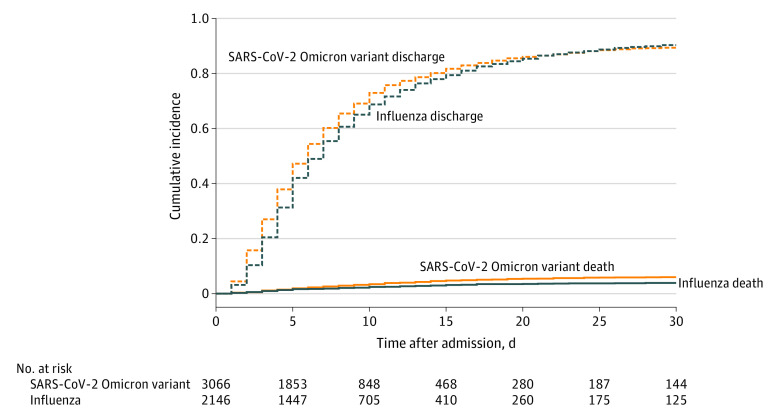
Cumulative Incidence Plot for Mortality Mortality with discharge as competing risk, by disease status (the SARS-CoV-2 Omicron variant vs influenza).

The cause-specific hazard ratio (csHR) of the SARS-CoV-2 Omicron variant vs influenza on in-hospital death was 1.93 (95% CI, 1.47- 2.54; *P* < .001), and the csHR for the competing risk hospital discharge was 1.21 (95% CI, 1.14-1.28; *P* < .001), suggesting that patients with the SARS-CoV-2 Omicron variant had a higher daily risk of mortality over a shorter period of hospital stay. These cause-specific risks can be summarized in the adjusted subdistribution hazard ratio (sdHR), showing an effect size of 1.54 (95% CI, 1.18-2.00; *P* = .002) on in-hospital mortality for the SARS-CoV-2 Omicron variant compared with influenza, while considering hospital discharge as a competing event for the SARS-CoV-2 Omicron variant compared with influenza. The corresponding cumulative incidence plot for death and the competing risk of discharge is shown in Figure 2.

**Figure 3.**
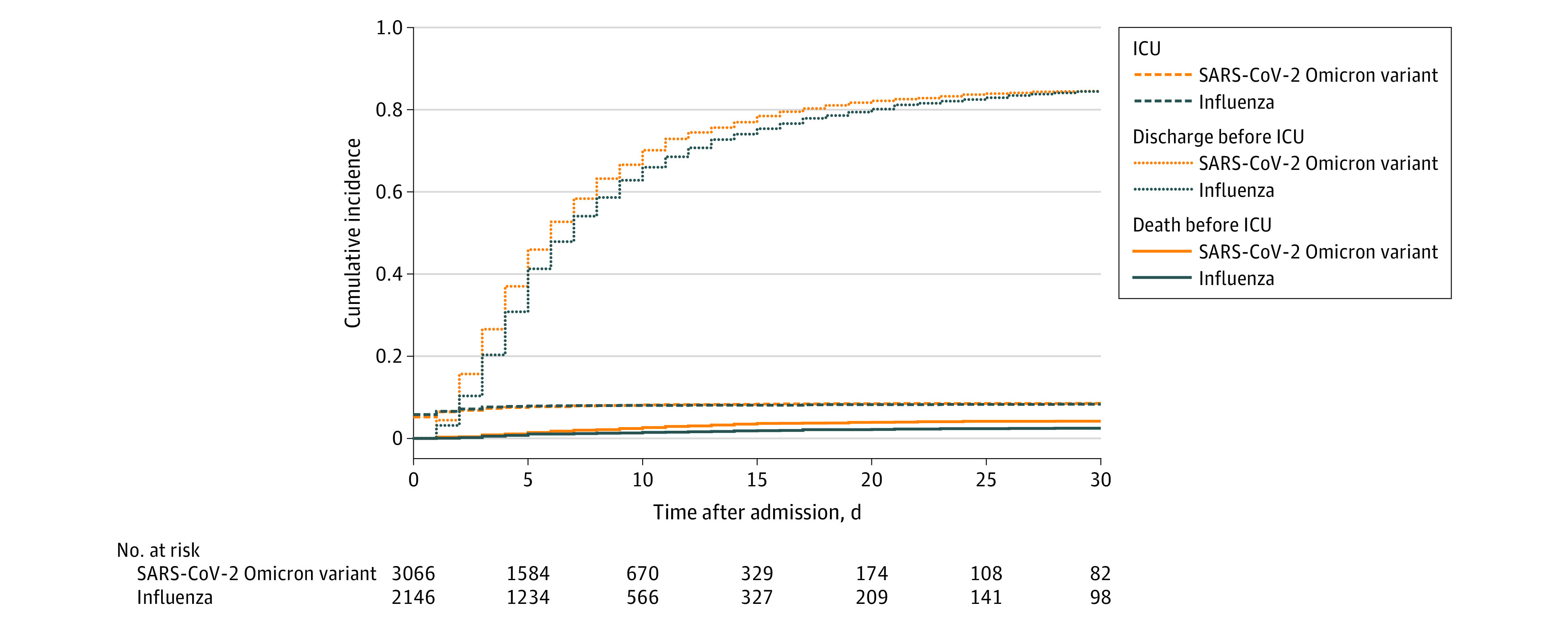
Cumulative Incidence Plot for Intensive Care Unit (ICU) Admission ICU admission with discharge and death before ICU admission as competing risk, by disease status (the SARS-CoV-2 Omicron variant vs influenza).

The csHR for ICU admission for patients with the SARS-CoV-2 Omicron variant vs influenza was 1.13 (95% CI, 0.91-1.39; *P* = .20), and for the competing risk of discharge before ICU admission, the csHR was 1.29 (95% CI, 1.21-1.37; *P* < .001). There was also a significantly higher rate of death (competing event) for patients with the SARS-CoV-2 Omicron variant than those with influenza (csHR, 2.48; 95% CI, 1.75-3.50; *P* < .001), indicating that patients with the SARS-CoV-2 Omicron variant had a higher daily risk of dying, or being discharged, which limited excess ICU admission rates. This resulted in a summary effect size, adjusted for confounders, of the SARS-CoV-2 Omicron variant for ICU admission of 1.08 (95% CI, 0.88-1.32, *P* = .50) (Figure 3).

eTable 5 in Supplement 1 provides an overview of the comparison between the 160 patients with the SARS-CoV-2 Omicron variant who died before ICU admission and the 54 patients with the SARS-CoV-2 Omicron variant who died after ICU admission. Older age (median [IQR], 85 [75-90] years vs 71 [64-79] years; *P* < .001) and presence of dementia (33 of 160 [22.8%] vs 3 of 54 [5.6%]; *P* = .009) were associated with death before ICU admission vs death after ICU admission.

### Further Analyses

Patients hospitalized primarily because of (and not with) COVID-19 or influenza were included in a subgroup analysis (1522 of 3066 patients with the SARS-CoV-2 Omicron variant [49.6%] and 516 of 2146 patients with influenza [24.0%]). eTable 6 in Supplement 1 shows baseline characteristics of these groups, and eTable 7 in Supplement 1 shows the crude clinical outcomes. eTable 8 in Supplement 1 provides an overview on the adjusted csHRs. The resulting, final sdHR of in-hospital death was 2.86 (95% CI, 1.64-4.97; *P* < .001) for the SARS-CoV-2 Omicron variant vs influenza (cumulative incidence appears in eFigure 1 in Supplement 1). In addition, the SARS-CoV-2 Omicron variant vs influenza was now significantly associated with ICU admission (sdHR, 1.69; 95% CI, 1.09-2.62; *P* = .02) (eFigure 2 in Supplement 1). In eTables 9 to 11 in Supplement 1, a similar analysis was additionally made, only including patients admitted to the hospital because of COVID-19 or influenza during the same time period (January 15 to March 15, 2022). The results were in the range of the results from eTable 8 in Supplement 1.

In a second subgroup analysis, we only included patients with the SARS-CoV-2 Omicron variant who had never been vaccinated (1056 [34.4%]). Baseline characteristics and crude clinical outcomes of this analysis are shown in eTables 12 and 13 in Supplement 1. eTable 14 in Supplement 1 provides the overview on the adjusted csHRs. The sdHR for in-hospital mortality in comparison with all patients with influenza was 2.04 (95% CI, 1.50-2.79; *P* < .001), and the sdHR for ICU admission was 1.42 (95% CI, 1.11-1.82; *P* = .009) (eFigures 3 and 4 in Supplement 1).

eTable 15 in Supplement 1 provides an additional survival analysis with the date of positive test result as inclusion date. eTables 16 to 18 in Supplement 1 show an analysis excluding patients with an origin other than home. The results were comparable with the main analysis.

## Discussion

### Key Results

In this study, the outcomes of hospitalized patients with the SARS-CoV-2 Omicron variant were compared with those of patients hospitalized with influenza. After accounting for competing events as well as imbalance between patient groups, a significant 1.5-fold higher risk of in-hospital mortality was observed for patients with the SARS-CoV-2 Omicron variant vs influenza A or B infections. The risk of ICU admission was not significantly higher with the SARS-CoV-2 Omicron variant vs influenza. However, the csHR for in-hospital death before ICU admission was 2 to 3 times higher with the SARS-CoV-2 Omicron variant than with influenza. Preliminary results indicated that older age and dementia were associated with death before ICU admission compared with death after ICU admission; this could indicate the role of dispensed ICU admission.

A subgroup analysis that included patients hospitalized primarily because of COVID-19 or influenza showed an approximately 2.5 higher hazard ratio of in-hospital mortality and 1.7 higher ratio for admission to the ICU with the SARS-CoV-2 Omicron variant vs influenza. This indicates that the association of the SARS-CoV-2 Omicron variant with increased mortality could be even stronger. The difference in these results could be explained by the fact that more asymptomatic the SARS-CoV-2 Omicron variant vs influenza cases may have been detected due to a widespread admission screening program for COVID-19 in the first quarter of 2022.

The main strengths of our study include the multicenter setting, with data from a well-established prospective registry as well as an adequate and sophisticated statistical method. Data were collected by trained professionals, and data quality checks are performed regularly. Many hospitals (mainly large ones, but also some smaller, rural hospitals) participated throughout Switzerland, increasing the external validity of the study, although this will depend on the comparability of the health care setting (vaccination policies, ICU availabilities, admission testing strategy).

### Interpretation

When we compare our results with the previous study (12.8% in-house COVID-19 mortality for wildtype and Alpha variant),^16^ the SARS-CoV-2 Omicron variant mortality is now lower (7.0%) but still substantially higher than influenza. Importantly, despite a high level of preexisting immunity in the Swiss population of 98%, vaccination still plays a significant role regarding the main outcome, as the hazard ratio for death in the subgroup of unvaccinated patients was even 2-fold higher when compared with patients with influenza.^23^ Our results demonstrate that COVID-19 still cannot simply be compared with influenza. The increased csHR for mortality before admission to the ICU is of concern. The question of why these severely ill patients were not admitted to the ICU should be further evaluated. Therefore, our study may underestimate the need for ICU treatment in patients with the SARS-CoV-2 Omicron variant.

In addition, within the analysis considering only patients hospitalized primarily because of the SARS-CoV-2 Omicron variant or influenza, we found not only a greater difference in mortality between patients with the SARS-CoV-2 Omicron variant and influenza, but also a higher risk of ICU admission for patients with Omicron. This suggests that our main results may underestimate the increased severity of Omicron outcomes, potentially due to a more stringent admission screening for the SARS-CoV-2 Omicron variant vs Influenza, therefore including some asymptomatic or oligosymptomatic patients in the cohort.^7,8,24^ With that in mind, it does not seem surprising that the CURB-65 score tended to be marginally higher for patients with influenza.^22^

Furthermore, the therapeutic options for the SARS-CoV-2 Omicron variant (including the stepwise introduction of nirmatrelvir in Switzerland) evolved during the study. Therefore, Omicron mortality may further decrease in the future.

### Limitations

There are limitations to this study. First, not all cases were sequenced, so variants other than Omicron could have been included in the study data. However, we only included patients admitted to the hospital from January 15, 2022, onwards, when more than 95% of all sequenced positive test results indicated the Omicron variant. From February 5, 2022, the percentage increased to more than 99%.^2^ This is also reflected in our cohort: in 469 sequenced samples, more than 95% were identified as Omicron. Second, there was very limited information on the vaccination type (mRNA vs live-attenuated vs protein based) for patients with the SARS-CoV-2 Omicron variant; therefore this variable could not be considered in analysis. Nevertheless, more than 95% of vaccinated patients in Switzerland were estimated to be vaccinated with an mRNA vaccine.^25^ In addition, not all centers completed follow-up information, resulting in a small proportion of missing outcomes and subsequent exclusions. However, we do not think that these limitations have introduced a major bias. We also cannot exclude the possibility that certain older patients with serious the SARS-CoV-2 Omicron variant remained in a nursing home and were not hospitalized at all, which could bias the in-hospital results. Additionally, as reported earlier in this article, there may have been diagnostic bias due to a very comprehensive screening for Omicron colonization. Therefore, supported by the subgroup analysis, our main results may underestimate the relative severity of true SARS-CoV-2 Omicron variant disease. In addition, information on influenza subtypes was largely lacking. However, in our additional analyses, we compared SARS-CoV-2 Omicron variant and influenza cases from the same time period (2022), with H3N2 dominating in Switzerland, and still found a higher mortality for the SARS-CoV-2 Omicron variant.^12^ Furthermore, we cannot exclude residual confounding, as information on influenza vaccination, preexisting immunity as well as further potential unidentified confounding variables were largely missing.

## Conclusions

This study shows that the COVID-19 due to the Omicron variant was associated with a higher risk of in-hospital mortality compared with patients with influenza. This indicates that the SARS-CoV-2 Omicron variant should still be taken seriously, and improved prevention and treatment strategies are still highly relevant, although overburdening of the health care system has become less likely over time.

## Notes

Supplement 1. **eTable 1.** Balance Check of Inverse Probability Weights **eTable 2.** Scaled Schoenfeld Residuals to Test for Proportional Hazards Assumption **eTable 3.** Detailed Compilation of Different Variants of Sequenced COVID-19 Samples **eTable 4.** Overview of the CURB-65 Severity Score at Admission **eTable 5.** Baseline Characteristics of Patients Died With Omicron (Admitted January 15 to March 15, 2022) With and Without Intensive Care Unit Treatment, Switzerland **eTable 6.** Baseline Characteristics of Patients With Omicron (Admitted January 15 to March 15, 2022) and Influenza A or B (Admitted 2022) Hospitalized Because of Omicron/Influenza, Switzerland (n=2038) **eTable 7.** Unadjusted Crude Outcomes of Patients With Omicron (Admitted January 15 to March 15, 2022) and Influenza A or B (Admitted 2022), Hospitalized Because of COVID-19 or Influenza, Switzerland (n=2038) **eTable 8.** Outcomes of Patients With Omicron (Admitted January 15 to March 15, 2022) and Influenza A or B (Admitted 2022) Hospitalized Because of COVID-19 or Influenza, Switzerland (n=2038) **eTable 9.** Baseline Characteristics of Patients Hospitalized Only Because of Omicron (Admitted January 15 to March 15, 2022) and Because of Influenza A/B (Admitted January 15 to March 15, 2022), Switzerland (n=2010) **eTable 10.** Unadjusted Crude Clinical Outcomes of Patients Admitted Only Because of Omicron (Admitted January 15 to March 15, 2022) and Because of Influenza A or B Patients (Admitted January 15 to March 15, 2022), Switzerland (n=2010) **eTable 11.** Clinical Outcomes of Patients Admitted Only Because of Omicron (Admitted January 15 to March 15, 2022) and Because of Influenza A or B Patients (Admitted January 15 to March 15, 2022), Switzerland (n=2010) **eTable 12.** Baseline Characteristics of Unvaccinated Patients With Omicron (Admitted January 15 to March 15, 2022) and All Influenza A or B Patients (Admitted 2018-2022), Switzerland (n=3202) **eTable 13.** Unadjusted Crude Clinical Outcomes of Unvaccinated Patients With Omicron (Admitted January 15 to March 15, 2022) and All Influenza A or B Patients (Admitted 2018-2022), Switzerland (n=3202) **eTable 14.** Clinical Outcomes of Unvaccinated Patients With Omicron (Admitted January 15 to March 15, 2022) and All Influenza A or B Patients (Admitted 2018-2022), Switzerland (n=3202) **eTable 15.** Clinical Outcomes of Patients With Omicron (Admitted January 15 to March 15, 2022) and All Influenza A or B Patients (Admitted 2018-2022), Switzerland, With Date of Positive Test Result as Inclusion Date (n=5193) **eTable 16.** Baseline Characteristics of Patients With Omicron (Admitted January 15 to March 15, 2022) and Influenza A or B Patients (Admitted 2018-2022); Transfers From LTCF Origin and Referring Hospitals Excluded, Switzerland (n=4675) **eTable 17.** Unadjusted Crude Clinical Outcomes of Patients With Omicron (Admitted January 15 to March 15, 2022) and Influenza A or B Patients (Admitted January 15 to March 15, 2022); Transfers From LTCF Origin and Referring Hospitals Excluded, Switzerland (n=4675) **eTable 18.** Clinical Outcomes of Patients With Omicron (Admitted January 15 to March 15, 2022) and All Influenza A or B (Admitted 2018-2022), LTCF Origin and Others Excluded, Switzerland (n=4675) **eFigure 1.** Cumulative Incidence Plot for Mortality for Patients Because of SARS-CoV-2 Omicron Variant or Influenza **eFigure 2.** Cumulative Incidence Plot for ICU for Patients Because of SARS-CoV-2 Omicron Variant or Influenza **eFigure 3.** Cumulative Incidence Plot for Morality Among Patients with SARS-CoV-2 Omicron Variant Who Were Unvaccinated and Patients with Influenza **eFigure 4.** Cumulative Incidence Plot for ICU Admission Among Patients with SARS-CoV-2 Omicron Variant Who Were Unvaccinated and Patients with Influenza
eReferences.

Supplement 2.
Nonauthor Collaborators

Supplement 3.
Data Sharing Statement
