# Supplementary material for: Hospital Outcomes of Community-Acquired SARS-CoV-2 Omicron Variant Infection Compared With Influenza Infection in Switzerland
Source: JAMA Netw Open. 2023 Feb 15;6(2):e2255599. doi: 10.1001/jamanetworkopen.2022.55599 (PMC9932839; doi:10.1001/jamanetworkopen.2022.55599)
Supplement: Supplement 1. — eTable 1. Balance Check of Inverse Probability Weights eTable 2. Scaled Schoenfeld Residuals to Test for Proportional Hazards Assumption eTable 3. Detailed Compilation of Different Variants of Sequenced COVID-19 Samples eTable 4. Overview of the CURB-65 Severity Score at Admission eTable 5. Baseline Characteristics of Patients Died With Omicron (Admitted January 15 to March 15, 2022) With and Without Intensive Care Unit Treatment, Switzerland eTable 6. Baseline Characteristics of Patients With Omicron (Admitted January 15 to March 15, 2022) and Influenza A or B (Admitted 2022) Hospitalized Because of Omicron/Influenza, Switzerland (n=2038) eTable 7. Unadjusted Crude Outcomes of Patients With Omicron (Admitted January 15 to March 15, 2022) and Influenza A or B (Admitted 2022), Hospitalized Because of COVID-19 or Influenza, Switzerland (n=2038) eTable 8. Outcomes of Patients With Omicron (Admitted January 15 to March 15, 2022) and Influenza A or B (Admitted 2022) Hospitalized Because of COVID-19 or Influenza, Switzerland (n=2038) eTable 9. Baseline Characteristics of Patients Hospitalized Only Because of Omicron (Admitted January 15 to March 15, 2022) and Because of Influenza A/B (Admitted January 15 to March 15, 2022), Switzerland (n=2010) eTable 10. Unadjusted Crude Clinical Outcomes of Patients Admitted Only Because of Omicron (Admitted January 15 to March 15, 2022) and Because of Influenza A or B Patients (Admitted January 15 to March 15, 2022), Switzerland (n=2010) eTable 11. Clinical Outcomes of Patients Admitted Only Because of Omicron (Admitted January 15 to March 15, 2022) and Because of Influenza A or B Patients (Admitted January 15 to March 15, 2022), Switzerland (n=2010) eTable 12. Baseline Characteristics of Unvaccinated Patients With Omicron (Admitted January 15 to March 15, 2022) and All Influenza A or B Patients (Admitted 2018-2022), Switzerland (n=3202) eTable 13. Unadjusted Crude Clinical Outcomes of Unvaccinated Patients With Omicron (Admitted Janua [file jamanetwopen-e2255599-s001.pdf]

Supplemental Online Content

Portmann L, de Kraker MEA, Fröhlich G, et al; CH-SUR study group. Hospital outcomes of community-acquired SARS-CoV-2 Omicron variant infection compared with influenza infection in Switzerland. *JAMA Netw Open.* 2023;6(2):e2255599. doi:10.1001/jamanetworkopen.2022.55599

eTable 1. Balance Check of Inverse Probability Weights

eTable 2. Scaled Schoenfeld Residuals to Test for Proportional Hazards Assumption

eTable 3. Detailed Compilation of Different Variants of Sequenced COVID-19 Samples

eTable 4. Overview of the CURB-65 Severity Score at Admission

eTable 5. Baseline Characteristics of Patients Died With Omicron (Admitted January 15 to March 15, 2022) With and Without Intensive Care Unit Treatment, Switzerland

eTable 6. Baseline Characteristics of Patients With Omicron (Admitted January 15 to March 15, 2022) and Influenza A or B (Admitted 2022) Hospitalized Because of Omicron/Influenza, Switzerland (n=2038)

eTable 7. Unadjusted Crude Outcomes of Patients With Omicron (Admitted January 15 to March 15, 2022) and Influenza A or B (Admitted 2022), Hospitalized Because of COVID-19 or Influenza, Switzerland (n=2038)

eTable 8. Outcomes of Patients With Omicron (Admitted January 15 to March 15, 2022) and Influenza A or B (Admitted 2022) Hospitalized Because of COVID-19 or Influenza, Switzerland (n=2038)

eTable 9. Baseline Characteristics of Patients Hospitalized Only Because of Omicron (Admitted January 15 to March 15, 2022) and Because of Influenza A/B (Admitted January 15 to March 15, 2022), Switzerland (n=2010)

eTable 10. Unadjusted Crude Clinical Outcomes of Patients Admitted Only Because of Omicron (Admitted January 15 to March 15, 2022) and Because of Influenza A or B Patients (Admitted January 15 to March 15, 2022), Switzerland (n=2010)

eTable 11. Clinical Outcomes of Patients Admitted Only Because of Omicron (Admitted January 15 to March 15, 2022) and Because of Influenza A or B Patients (Admitted January 15 to March 15, 2022), Switzerland (n=2010)

eTable 12. Baseline Characteristics of Unvaccinated Patients With Omicron (Admitted January 15 to March 15, 2022) and All Influenza A or B Patients (Admitted 2018-2022), Switzerland (n=3202)

eTable 13. Unadjusted Crude Clinical Outcomes of Unvaccinated Patients With Omicron (Admitted January 15 to March 15, 2022) and All Influenza A or B Patients (Admitted 2018-2022), Switzerland (n=3202)

eTable 14. Clinical Outcomes of Unvaccinated Patients With Omicron (Admitted January 15 to March 15, 2022) and All Influenza A or B Patients (Admitted 2018-2022), Switzerland (n=3202)

eTable 15. Clinical Outcomes of Patients With Omicron (Admitted January 15 to March 15, 2022) and All Influenza A or B Patients (Admitted 2018-2022), Switzerland, With Date of Positive Test Result as Inclusion Date (n=5193)

© 2023 Portmann L et al. *JAMA Network Open.*

eTable 16. Baseline Characteristics of Patients With Omicron (Admitted January 15 to March 15, 2022) and Influenza A or B Patients (Admitted 2018-2022); Transfers From LTCF Origin and Referring Hospitals Excluded, Switzerland (n=4675)

eTable 17. Unadjusted Crude Clinical Outcomes of Patients With Omicron (Admitted January 15 to March 15, 2022) and Influenza A or B Patients (Admitted January 15 to March 15, 2022); Transfers From LTCF Origin and Referring Hospitals Excluded, Switzerland (n=4675)

eTable 18. Clinical Outcomes of Patients With Omicron (Admitted January 15 to March 15, 2022) and All Influenza A or B (Admitted 2018-2022), LTCF Origin and Others Excluded, Switzerland (n=4675)

eFigure 1. Cumulative Incidence Plot for Mortality for Patients Because of SARS-CoV-2 Omicron Variant or Influenza

eFigure 2. Cumulative Incidence Plot for ICU for Patients Because of SARS-CoV-2 Omicron Variant or Influenza

eFigure 3. Cumulative Incidence Plot for Morality Among Patients with SARS-CoV-2 Omicron Variant Who Were Unvaccinated and Patients with Influenza

eFigure 4. Cumulative Incidence Plot for ICU Admission Among Patients with SARS-CoV-2 Omicron Variant Who Were Unvaccinated and Patients with Influenza

eReferences.

This supplemental material has been provided by the authors to give readers additional information about their work.

© 2023 Portmann L et al. *JAMA Network Open.*

eTable 1. Balance Check of Inverse Probability Weights

We used the same methodology as described before[1] “We used balance.IPW from the CausalGAM package[2]: This function calculates weighted means of covariates and then examines the differences in the weighted means across influenza and COVID-19 cases as a diagnostic for covariate balance for inverse probability weighting.

The standardized mean differences between all variables in the dataset are reported along with a z-statistics for these standardized differences, whereby z-statistics closer to 0 imply better univariate mean balance.

The columns are (from left to right) the observed mean of the covariate among the COVID19 patients, the observed mean of the covariate among the influenza patients, the weighted mean of the covariate among the COVID-19 patients, the weighted mean of the covariate among the influenza patients, the weighted mean difference, and the z-statistic for the difference.

The formula (logistic regression with logit-link) used for calculation of weights was: Virus type ~ Age + Gender + Admission to University Hospital”[1]

	obs.mean.t	obs.mean.c	w.mean.t	w.mean.c	w.mean.diff	z
age	66.037	69.180	67.185	67.101	0.083	1.100
Gender (0=male, 1=female	0.484	0.519	0.499	0.500	-0.001	0.442
University hospital (0=no, 1=yes)	0.356	0.628	0.466	0.466	0.001	1.237

© 2023 Portmann L et al. *JAMA Network Open.*

eTable 2. Scaled Schoenfeld Residuals to Test for Proportional Hazards Assumption[3]

	Chi-Square	p
In Hospital Death		
csHR[a] Death	1.86	0.17
csHR[a] Discharge	22.1	<0.001
sdHR[b] Death	0.708	0.4
ICU admission		
csHR[a] ICU admission	7.26	0.007
csHR[a] Death before ICU	0.0679	0.79
csHR[a] Discharge before ICU	0.708	0.4
sdHR[b] ICU admission	3.41	0.07

[a]csHR: Cox specific hazard ratio, [b]sdHR: subdistribution hazard ratio

© 2023 Portmann L et al. *JAMA Network Open.*

eTable 3. Detailed Compilation of Different Variants of Sequenced COVID-19 Samples

	Number of sequenced samples (n=469)
Virus type	
Common variant/Mutation but no VOC (Variant of Concern)	2
A.23.1	1
B.1.617.2 - Delta	4
B.1.1.7 - Alpha	1
B.1.1	2
B.1.1.39	1
B.1	1
AY.46.6	2
AY.43	2
B.1.1.529/BA.1 - Omicron	283 (60.3%)
B.1.1.529/BA.2 – Omicron	73 (15.5%)
BA.1.1 - Omicron	93 (19.8%)
Other	4

© 2023 Portmann L et al. *JAMA Network Open.*

eTable 4. Overview of the CURB-65 Severity Score at Admission

	Covid-omicron (n=3066)	Influenza A/B (n=2146)
CURB-65-Score		
0	1063 (34.7)	186 (26.3)
1	1200 (39.1)	326 (46.2)
2	564 (18.4)	141 (20.0)
3	201 (6.6)	44 (6.2)
4	35 (1.1)	9 (1.3)
5	3 (0.1)	0 (0.0)

Missingness for Influenza: n=1440 (67.1%)

© 2023 Portmann L et al. *JAMA Network Open.*

eTable 5. Baseline Characteristics of Patients Died With Omicron (Admitted January 15 to March 15, 2022) With and Without Intensive Care Unit Treatment, Switzerland

	Omicron died with ICU (n=54)	Omicron died without ICU (n=160)	p
Age median in years (IQR)	71 [64.25, 79]	84.5 [74.75, 90]	<0.001
Sex			
Female, n (%)	16 (29.6)	57 (35.6)	0.52
Admission to university hospital, n (%)	15 (27.8)	47 (29.4)	0.96
BMI kg/m^2 median (IQR)	25.5 [23.45, 29.75][a]	24.46 [20.65, 27.7][b]	0.02
Immune state Covid			
Vaccinated, n (%)	24 (46.2)[c]	78 (52.3)[d]	0.54
Origin prior to hospitalization			0.42
Domicile, n (%)	47 (87)	136 (86.6)[e]	
LTC facility, n (%)	3 (5.6)	15 (9.6)[e]	
Other hospital	4 (7.4)	5 (3.2)[e]	
Comorbidities, n (%)	52 (96.3)	144 (98)[f]	0.87
Diabetes mellitus	19 (35.2)	32 (21.8)[f]	0.08
Chronic cardiovascular disease	27 (51.9)[g]	84 (57.5)[f]	0.59
Chronic kidney disease	19 (35.2)	50 (34.2)[f]	>0.99
Chronic pulmonary disease	12 (22.2)	33 (22.6)[f]	>0.99
Chronic neurologic impairment	5 (9.3)	22 (15.0)[f]	0.41
Haematological disorder	1 (1.9)	3 (2)[f]	>0.99
Chronic liver disease	7 (13)	13 (8.9)[f]	0.56
Dementia	3 (5.6)	33 (22.8)[f]	0.009

(n=214)

ICU: Intensive care unit, BMI: body mass index, IQR: interquartile range, LTC: Long-term care

Missing values: [a]15 (27.8%), [b]41 (25.6%), [c] 2 (3.6%), [d] 11 (6.8%) [e]3(1.9%), [f] 8.1-9.4% (missing mainly because one center did not report comorbidities), [g] 2(3.7%)

© 2023 Portmann L et al. *JAMA Network Open.*

eTable 6. Baseline Characteristics of Patients With Omicron (Admitted January 15 to March 15, 2022) and Influenza A or B (Admitted 2022) Hospitalized Because of Omicron/Influenza, Switzerland (n=2038)

	Covid omicron (n=1522)	Influenza A/B) (n=516)	p
Age median in years (IQR)	75 [61, 84]	74 [61, 84]	0.31
Sex			
Female, n (%)	701 (46.1)	268 (51.9)	0.02
Admission to university hospital, n (%)	380 (25)	248 (48.1)	<0.001
Comorbidities, n (%)*	1294 (86.9)	434 (89.3)	0.19
Diabetes mellitus	332 (22.3)	122 (25.1)	0.23
Chronic cardiovascular disease	602 (40.6)	177 (36.4)	0.11
Chronic kidney disease	373 (25.1)	86 (17.7)	0.001
Chronic pulmonary disease	239 (16.1)	128 (26.4)	<0.001
Chronic neurologic impairment	173 (11.6)	71 (14.7)	0.1
Haematological disorder	61 (4.1)	15 (3.1)	0.38
Chronic liver disease	42 (2.8)	20 (4.1)	0.2
Dementia	146 (9.9)	46 (9.6)	0.89

BMI: body mass index, IQR: interquartile range

Missing values: *for Covid and Influenza: Between 2.2-7.0% (missing mainly because one center did not report comorbidities).

© 2023 Portmann L et al. *JAMA Network Open.*

eTable 7. Unadjusted Crude Outcomes of Patients With Omicron (Admitted January 15 to March 15, 2022) and Influenza A or B (Admitted 2022), Hospitalized Because of COVID-19 or Influenza, Switzerland (n=2038)

	Covid omicron (n=1522)	Influenza A/B) (n=516)	p
In-hospital deaths, n (%)	129 (8.5)	17 (3.3)	<0.001
Admission to the ICU, n (%)	136 (9.2)[a]	27 (5.6)[b]	0.02
Length of ICU stay in days, median (IQR)	6 [2, 19]	3 [2, 6.5]	0.02
Invasive ventilation, n (%)	73 (53.7)	8 (29.6)	0.04
Length of hospital stay in days, median (IQR)	6 [4, 11]	5 [4, 10]	0.13
Complications, n (%)[c]	1223 (82.3)	378 (77.8)	0.03
Respiratory complications (%)	958 (64.7)	316 (65)	0.94
Cardiac disease (%)	183 (12.4)	77 (15.9)	0.061
Neurologic impairment (%)	46 (3.1)	21 (4.3)	0.26
Renal impairment (%)	212 (14.3)	70 (14.4)	>0.99
Antibiotic treatment (%)	486 (33)	221 (45.5)	<0.001

ICU: intensive care unit, IQR: interquartile range

Missing values: [a] 37 (2.4%) [b] 30 (5.8%) [c] for Covid and Influenza: Between 2.4-7% (missing mainly because one center did not report complications).

© 2023 Portmann L et al. *JAMA Network Open.*

eTable 8. Outcomes of Patients With Omicron (Admitted January 15 to March 15, 2022) and Influenza A or B (Admitted 2022) Hospitalized Because of COVID-19 or Influenza, Switzerland (n=2038)

	csHR[a]/sdHR[b]	Lower 95%CI[c]	Upper 95% CI[c]	p
In-Hospital-Death				
csHR[a] Death	2.83	1.60	4.97	<0.001
csHR[a] Discharge	0.92	0.82	1.04	0.20
sdHR[b] Death	2.86	1.64	4.98	<0.001
ICU admission				
csHR[a] ICU admission	1.69	1.08	2.63	0.02
csHR[a] Death before ICU	3.07	1.59	5.94	<0.001
csHR[a] Discharge before ICU	0.98	0.87	1.12	0.90
sdHR[b] ICU admission	1.68	1.08	2.61	0.02

[a]csHR: Cox specific hazard ratio, [b]sdHR: subdistribution hazard ratio, CI: Confidence Interval

© 2023 Portmann L et al. *JAMA Network Open.*

eTable 9. Baseline Characteristics of Patients Hospitalized Only Because of Omicron (Admitted January 15 to March 15, 2022) and Because of Influenza A/B (Admitted January 15 to March 15, 2022), Switzerland (n=2010)

	Covid omicron (n=1522)	Influenza A/B (n= 488)	p
Age median in years (IQR)	75 [61, 84]	74 [61, 84]	0.45
Sex			
Female, n (%)	701 (46.1)	253 (51.8)	0.03
Male, n(%)			
Admission to university hospital, n (%)	380 (25.0)	228 (46.7)	<0.001
Comorbidities, n (%)*	1294 (86.9)	408 (89.1)	0.25
Diabetes mellitus	332 (22.3)	110 (24.0)	0.48
Chronic cardiovascular disease	602 (40.6)	167 (36.5)	0.13
Chronic kidney disease	373 (25.1)	78 (17.1)	<0.001
Chronic pulmonary disease	239 (16.1)	119 (26.0)	<0.001
Chronic neurologic impairment	173 (11.6)	68 (14.9)	0.08
Haematological disorder	61 (4.1)	14 (3.1)	0.38
Chronic liver disease	42 (2.8)	19 (4.2)	0.20

BMI: body mass index, IQR: interquartile range

Missing values: *for Covid and Influenza: Between 2.2-6.6% (missing mainly because one center did not report comorbidities).

© 2023 Portmann L et al. *JAMA Network Open.*

eTable 10. Unadjusted Crude Clinical Outcomes of Patients Admitted Only Because of Omicron (Admitted January 15 to March 15, 2022) and Because of Influenza A or B Patients (Admitted January 15 to March 15, 2022), Switzerland (n=2010)

	Covid-omicron (n=1522)	Influenza A/B (n= 488)	p
In-hospital deaths, n (%)	129 (8.5)	16 (3.3)	<0.001
Deaths caused by Covid/Influenza	110 (7.3)[d]	11 (2.3)[e]	<0.001
Admission to the ICU, n (%)	136 (9.2)[a]	24 (5.2)[b]	0.01
Length of ICU stay in days, median (IQR)	6 [2, 19]	3.5 [2, 7]	0.04
Invasive ventilation, n (%)	73 (53.7)	7 (29.2)	0.05
Length of hospital stay in days, median (IQR)	6 [4, 11]	5 [4, 10]	0.12
Complications, n (%)[c]	1223 (82.3)	357 (77.9)	0.04
Respiratory complications	958 (64.7)	299 (65.3)	0.86
Cardiac disease	183 (12.4)	70 (15.3)	0.13
Neurologic impairment	46 (3.1)	20 (4.4)	0.25
Renal impairment	212 (14.3)	66 (14.4)	1
Antibiotic treatment	486 (33.0)	206 (45.0)	<0.001

ICU: intensive care unit, IQR: interquartile range

Missing values: [a] 37 (2.4%) [b] 30 (6.1%) [c] for Covid and Influenza: Between 2.7-6.4% (missing mainly because one center did not report complications).[d], 9 (0.6%), [e] 4 (0.8%)

© 2023 Portmann L et al. *JAMA Network Open.*

eTable 11. Clinical Outcomes of Patients Admitted Only Because of Omicron (Admitted January 15 to March 15, 2022) and Because of Influenza A or B Patients (Admitted January 15 to March 15, 2022), Switzerland (n=2010)

	csHR[a]/sdHR[b]	Lower 95% CI[c]	Upper 95% CI[c]	p
In-Hospital-Death				
csHR[a] Death	2.93	1.64	5.20	<0.001
csHR[a] Discharge	0.93	0.83	1.05	0.2
sdHR[b] Death	2.93	1.66	5.18	<0.001
ICU admission				
csHR[a] ICU admission	1.79	1.12	2.84	0.01
csHR[a] Death before ICU	3.29	1.68	6.46	<0.001
csHR[a] Discharge before ICU	0.99	0.87	1.13	0.9
sdHR[b] ICU admission	1.78	1.12	2.82	0.01

[a]csHR: Cox specific hazard ratio, [b]sdHR: subdistribution hazard ratio, CI: Confidence Interval

© 2023 Portmann L et al. *JAMA Network Open.*

eTable 12. Baseline Characteristics of Unvaccinated Patients With Omicron (Admitted January 15 to March 15, 2022) and All influenza A or B Patients (Admitted 2018-2022), Switzerland (n=3202)

	Unvaccinated Covid omicron (n=1056)	Influenza A/B (n= 2146)	p
Age median in years (IQR)	70 [53, 83]	73.5 [59.25, 83]	<0.001
Sex			
Female, n (%)	546 (51.7)	1113 (51.9)	0.96
Admission to university hospital, n (%)	363 (34.4)	1347 (62.8)	<0.001
Comorbidities, n (%)*	750 (76.5)	1648 (80.9)	0.006
Diabetes mellitus	171 (17.5)	451 (22.2)	0.003
Chronic cardiovascular disease	327 (33.5)	747 (36.7)	0.10
Chronic kidney disease	185 (18.9)	368 (18.1)	0.62
Chronic pulmonary disease	108 (11.1)	499 (24.6)	<0.001
Chronic neurologic impairment	90 (9.2)	292 (14.4)	<0.001
Haematological disorder	20 (2)	182 (8.9)	<0.001
Chronic liver disease	22 (2.2)	92 (4.5)	0.003

BMI: body mass index, IQR: interquartile range

Missing values: *for Covid and Influenza: Between 5.1-8% (missing mainly because one center did not report comorbidities).

© 2023 Portmann L et al. *JAMA Network Open.*

eTable 13. Unadjusted Crude Clinical Outcomes of Unvaccinated Patients With Omicron (Admitted January 15 to March 15, 2022) and All Influenza A or B Patients (Admitted 2018-2022), Switzerland (n=3202)

	Unvaccinated Covid omicron (n=1056)	Influenza A/B (n=2146)	p
In-hospital deaths, n (%)	99 (9.4)	95 (4.4)	<0.001
Admission to the ICU, n (%)	110 (11.1)[a]	169 (8.3)[b]	0.02
Length of ICU stay in days, median (IQR)	5 [2, 18.5]	4 [2, 8.5]	0.006
Invasive ventilation, n (%)	59 (54.1)	77 (46.1)	0.24
Length of hospital stay in days, median (IQR)	6 [3, 11]	6 [4, 12]	0.03
Complications, n (%)*	709 (70.9)	1365 (67.3)	0.05
Respiratory complications	565 (56.9)	1142 (56.3)	0.79
Cardiac disease	99 (10.4)	310 (15.3)	<0.001
Neurologic impairment	31 (3.3)	110 (5.4)	0.01
Renal impairment	125 (13.0)	232 (11.4)	0.23
Antibiotic treatment	257 (26.7)	1160 (57.1)	<0.001

ICU: intensive care unit, IQR: interquartile range

Missing values: [a] 63 (6%) [b] 113 (5.3%) [c] for Covid and Influenza: Between 5.3-9.9% (missing mainly because one center did not report complications).

© 2023 Portmann L et al. *JAMA Network Open.*

eTable 14. Clinical Outcomes of Unvaccinated Patients With Omicron (Admitted January 15 to March 15, 2022) and All Influenza A or B Patients (Admitted 2018-2022), Switzerland (n=3202)

	csHR[a]/sdHR[b]	Lower 95% CI[c]	Upper 95% CI[c]	p
In-Hospital-Death				
csHR[a] Death	2.32	1.70	3.18	<0.001
csHR[a] Discharge	1.08	0.99	1.17	0.08
sdHR[b] Death	2.04	1.50	2.79	<0.001
ICU admission				
csHR[a] ICU admission	1.47	1.14	1.88	0.003
csHR[a] Death before ICU	3.23	2.20	4.75	<0.001
csHR[a] Discharge before ICU	1.19	1.09	1.30	<0.001
sdHR[b] ICU admission	1.42	1.11	1.82	0.006

[a]csHR: Cox specific hazard ratio, [b]sdHR: subdistribution hazard ratio, CI: Confidence Interval

© 2023 Portmann L et al. *JAMA Network Open.*

eTable 15. Clinical Outcomes of Patients With Omicron (Admitted January 15 to March 15, 2022) and All Influenza A or B Patients (Admitted 2018-2022), Switzerland, With Date of Positive Test Result as Inclusion Date (n=5193)

	csHR[a]/sdHR[b]	Lower 95% CI[c]	Upper 95% CI[c]	p
In-Hospital-Death				
csHR[a] Death	1.59	1.21	2.09	0.001
csHR[a] Discharge	1.03	0.97	1.1	0.3
sdHR[b] Death	1.50	1.44	1.97	0.003
ICU admission				
csHR[a] ICU admission	1.09	0.89	1.35	0.4
csHR[a] Death before ICU	1.97	1.4	2.78	<0.001
csHR[a] Discharge before ICU	1.07	1.00	1.14	0.04
sdHR[b] ICU admission	1.06	0.87	1.31	0.6

[a]csHR: Cox specific hazard ratio, [b]sdHR: subdistribution hazard ratio, CI: Confidence Interval

© 2023 Portmann L et al. *JAMA Network Open.*

eTable 16. Baseline Characteristics of Patients With Omicron (Admitted January 15 to March 15, 2022) and Influenza A or B Patients (Admitted 2018-2022); Transfers From LTCF Origin and Referring Hospitals Excluded, Switzerland (n=4675)

	Covid omicron (n=2743)	Influenza A/B (n= 1932)	p
Age median in years (IQR)	70 [53, 82]	73 [59, 83]	<0.001
Sex			
Female, n (%)	1336 (48.7)	992 (51.3)	0.08
Male, n(%)			
Admission to university hospital, n (%)	951 (34.7)	1196 (61.9)	<0.001
Comorbidities, n (%)*	2050 (79.0)	1481 (80.6)	0.19
Diabetes mellitus	494 (19.1)	410 (22.3)	0.009
Chronic cardiovascular disease	914 (35.4)	658 (35.8)	0.81
Chronic kidney disease	540 (20.9)	313 (17.0)	0.002
Chronic pulmonary disease	341 (13.2)	459 (25.0)	<0.001
Chronic neurologic impairment	255 (9.9)	257 (14.0)	<0.001
Haematological disorder	92 (3.6)	160 (8.7)	<0.001
Chronic liver disease	85 (3.3)	83 (4.5)	0.04

BMI: body mass index, IQR: interquartile range

Missing values: *for Covid and Influenza: Between 4.9-6% (missing mainly because one center did not report comorbidities).

© 2023 Portmann L et al. *JAMA Network Open.*

eTable 17. Unadjusted Crude Clinical Outcomes of Patients With Omicron (Admitted January 15 to March 15, 2022) and Influenza A or B Patients (Admitted January 15 to March 15, 2022); Transfers From LTCF Origin and Referring Hospitals Excluded, Switzerland (n=4675)

	Covid-omicron (n=2743)	Influenza A/B (n= 1932)	p
In-hospital deaths, n (%)	183 (6.7)	73 (3.8)	<0.001
Admission to the ICU, n (%)	194 (7.4)[a]	128 (7.0)[b]	0.595
Length of ICU stay in days, median (IQR)	3[1, 11.75]	4.[1.25, 7]	0.471
Invasive ventilation, n (%)	86 (45.0)	54 (43.2)	0.839
Length of hospital stay in days, median (IQR)	5 [3, 10]	6[4, 12]	<0.001
Complications, n (%)[c]	1484 (56.6)	1229 (67.1)	<0.001
Respiratory complications	1099 (42.1)	1023 (55.9)	<0.001
Cardiac disease	212 (8.4)	273 (14.9)	<0.001
Neurologic impairment	59 (2.3)	96 (5.2)	<0.001
Renal impairment	233 (9.2)	203 (11.1)	0.043
Antibiotic treatment	543 (21.1)	1024 (55.9)	<0.001

ICU: intensive care unit, IQR: interquartile range

Missing values: [a] 136 (5%) [b] 97 (5%) [c] for Covid and Influenza: Between 5.2-7.9% (missing mainly because one center did not report complications).

© 2023 Portmann L et al. *JAMA Network Open.*

eTable 18. Clinical Outcomes of Patients With Omicron (Admitted January 15 to March 15, 2022) and All Influenza A or B (Admitted 2018-2022), LTCF Origin and Others Excluded, Switzerland (n=4675)

	csHR[a]/sdHR[b]	Lower 95% CI[c]	Upper 95% CI[c]	p
In-Hospital-Death				
csHR[a] Death	2.23	1.64	3.03	<0.001
csHR[a] Discharge	1.22	1.14	1.23	<0.001
sdHR[b] Death	1.73	1.28	2.34	<0.001
ICU admission				
csHR[a] ICU admission	1.23	0.98	1.56	0.08
csHR[a] Death before ICU	2.86	1.94	4.21	<0.001
csHR[a] Discharge before ICU	1.30	1.22	1.40	<0.001
sdHR[b] ICU admission	1.16	0.92	1.47	0.2

[a]csHR: Cox specific hazard ratio, [b]sdHR: subdistribution hazard ratio, CI: Confidence Interval

© 2023 Portmann L et al. *JAMA Network Open.*

eFigure 1. Cumulative Incidence Plot for Mortality for Patients Because of COVID-19 Omicron or Influenza

Mortality with discharge as competing risk, by disease status (Covid-omicron versus influenza) for patients with omicron (admitted 15.01.2022-15.03.2022) and influenza A/B (admitted 2022) hospitalized *because* of omicron/influenza

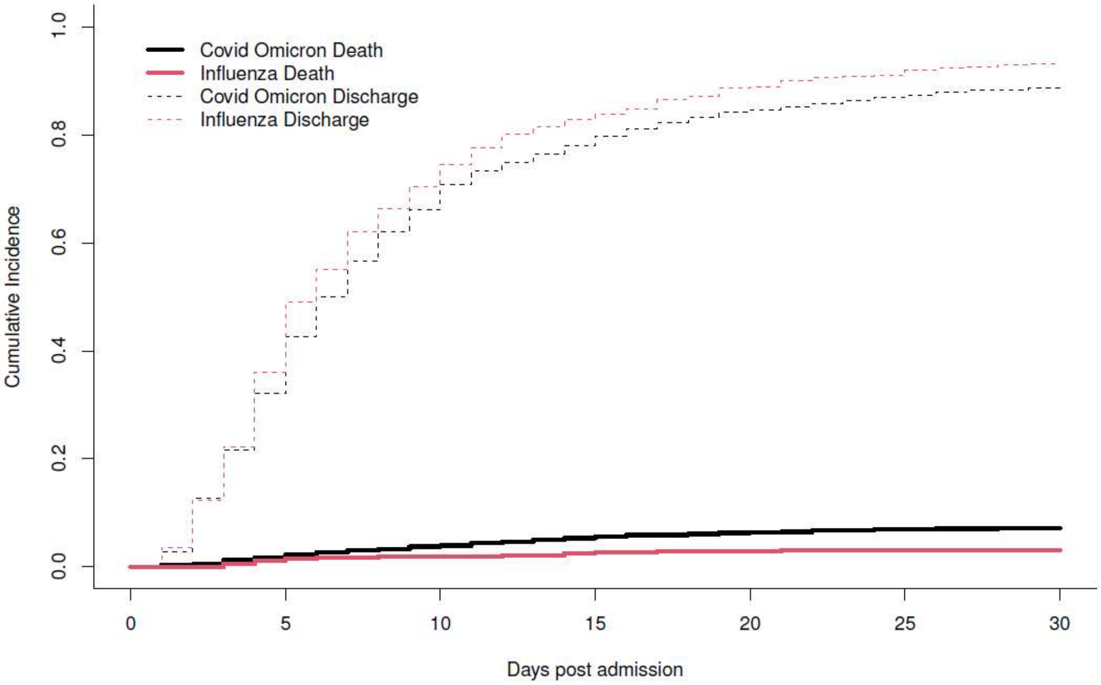

© 2023 Portmann L et al. *JAMA Network Open.*

eFigure 2. Cumulative Incidence Plot for ICU for Patients Because of COVID-19 Omicron or Influenza

ICU admission with discharge and death before ICU admission as competing risk, by disease status (Covid-omicron versus Influenza) for patients with omicron (admitted 15.01.2022-15.03.2022) and influenza A/B (admitted 2022) hospitalized *because* of omicron/influenza

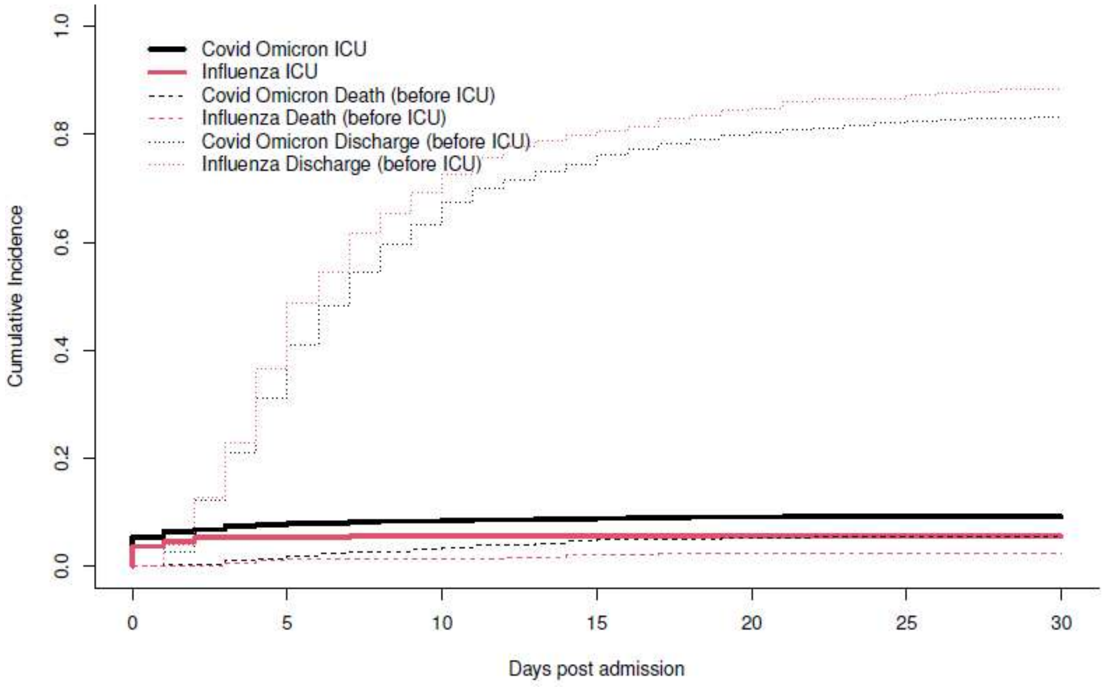

© 2023 Portmann L et al. *JAMA Network Open.*

eFigure 3. Cumulative Incidence Plot for Morality Among Patients with COVID-19 Omicron Who Were Unvaccinated and Patients with Influenza

Mortality with discharge as competing risk, by disease status (Covid-omicron versus influenza) for all unvaccinated patients with omicron (admitted 15.01.2022-15.03.2022) and all influenza A/B patients (admitted 2018-2022)

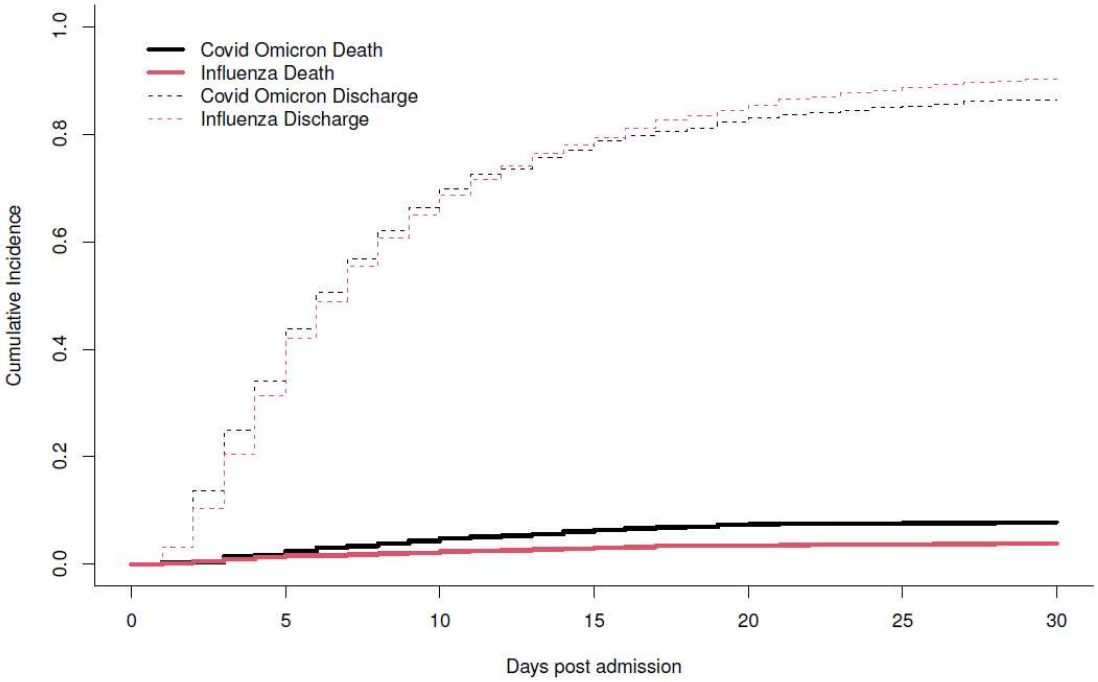

© 2023 Portmann L et al. *JAMA Network Open.*

eFigure 4. Cumulative Incidence Plot for ICU Admission Among Patients with COVID-19 Omicron Who Were Unvaccinated and Patients with Influenza

ICU admission with discharge and death before ICU admission as competing risk, by disease status (Covid-omicron versus Influenza) for all unvaccinated patients with omicron (admitted 15.01.2022-15.03.2022) and all influenza A/B patients (admitted 2018-2022)

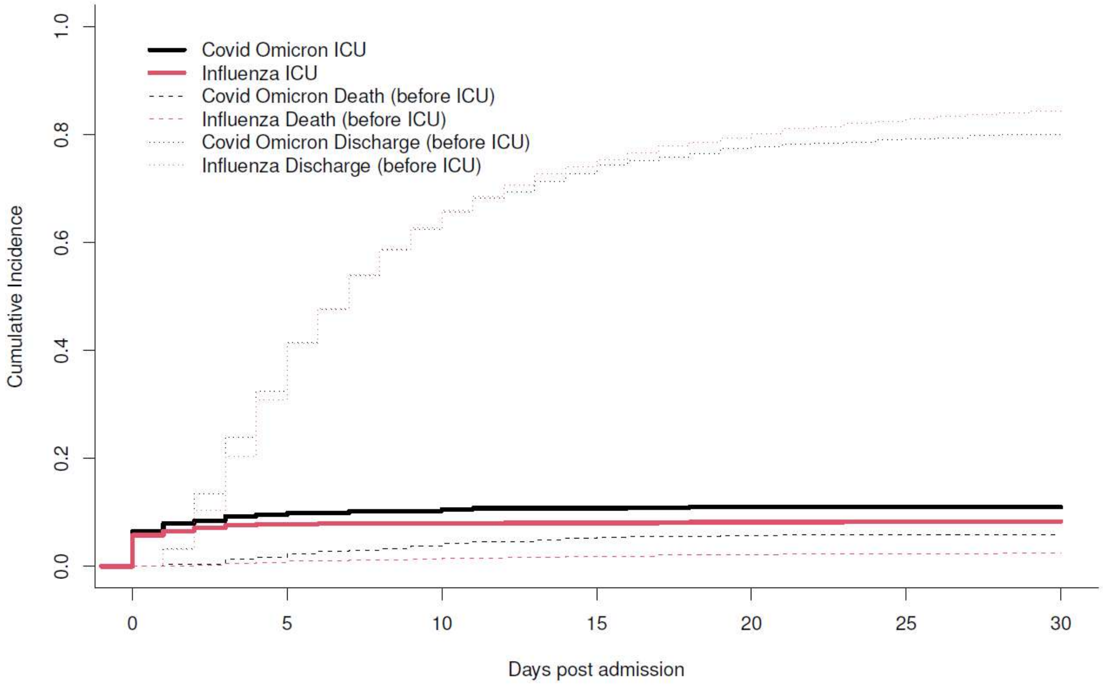

© 2023 Portmann L et al. *JAMA Network Open.*

eReferences

1. Fröhlich GM, Kraker MEA de, Abbas M, et al. Hospital outcomes of community-acquired COVID-19 versus influenza: Insights from the Swiss hospital-based surveillance of influenza and COVID-19. Euro Surveill. 2022;27(1). doi:10.2807/1560-7917.ES.2022.27.1.2001848.

2. Comprehensive R Archive Network (CRAN). CausalGAM: Estimation of Causal Effects with Generalized Additive Models. https://cran.r-project.org/web/packages/CausalGAM/. Updated December 13, 2022. Accessed December 18, 2022.

3. R: Test the Proportional Hazards Assumption of a Cox Regression. https://stat.ethz.ch/R-manual/R-devel/library/survival/html/cox.zph.html. Updated November 17, 2022. Accessed December 18, 2022.

© 2023 Portmann L et al. *JAMA Network Open.*
