# Supplementary material for: Hospital Outcomes of Community-Acquired SARS-CoV-2 Omicron Variant Infection Compared With Influenza Infection in Switzerland
Source: JAMA Netw Open. 2023 Feb 15;6(2):e2255599. doi: 10.1001/jamanetworkopen.2022.55599 (PMC9932839; doi:10.1001/jamanetworkopen.2022.55599)
Supplement: Supplement 2. — Nonauthor Collaborators [file jamanetwopen-e2255599-s002.pdf]

Supplemental Online Content: Nonauthor Collaborators
*First name, last name, and suffix (if applicable) are required and will appear in PubMed.

*Group Name(s): CH-SUR							
***First Name and Middle Initial(s)**	***Last Name**	***Suffix (eg, Jr, III)**	Academic Degrees	Institution	Location (city, state/province, country)	Role or Contribution, eg, chair, principal investigator	Group (if more than 1 Group listed in the byline) and/or Subgroup (eg, Steering Committee)
Sarah	Tschudin-Sutter		Prof.	Basel University Hospital	Basel, Switzerland	CH-SUR Local PI	
Ulrich	Heininger		Prof.	Basel Children University Hospital	Basel, Switzerland	CH-SUR Local PI	
Philipp	Jent		Dr.	Bern University Hospital	Bern, Switzerland	CH-SUR Local PI	
Michael	Büttcher		Dr.	Lucerne Cantonal Hospital	Lucerne, Switzerland	CH-SUR Local PI	
Yvonne	Nussbaumer		Dr.	Schaffhausen Cantonal Hospital	Schaffhausen, Switzerland	CH-SUR Local PI	
Danielle	Vuichard Gysin		Dr.	Thurgau Cantonal Hospital	Münsterlingen, Switzerland	CH-SUR Local PI	
Christoph	Berger		Prof.	Zurich Children University Hospital	Zurich, Switzerland	CH-SUR Local PI	
Domenica	Flury		Dr.	St. Gallen Cantonal Hospital	St. Gallen, Switzerland	CH-SUR Local PI	
Marie-Céline	Zanella Terrier		Dr.	Geneve University Hospital	Geneve, Switzerland	CH-SUR Local PI	
