# Supplementary material for: Hospital Outcomes of Community-Acquired SARS-CoV-2 Omicron Variant Infection Compared With Influenza Infection in Switzerland
Source: JAMA Netw Open. 2023 Feb 15;6(2):e2255599. doi: 10.1001/jamanetworkopen.2022.55599 (PMC9932839; doi:10.1001/jamanetworkopen.2022.55599)
Supplement: Supplement 3. — Data Sharing Statement [file jamanetwopen-e2255599-s003.pdf]

Data Sharing Statement

Portmann. Hospital Outcomes of Community-Acquired SARS-CoV-2 Omicron Variant Infection Compared With Influenza Infection in Switzerland. *JAMA Netw Open*. Published February 15, 2023. doi:10.1001/jamanetworkopen.2022.55599

Data
Data available: No
